# Molecular features in young vs elderly breast cancer patients and the impacts on survival disparities by age at diagnosis

**DOI:** 10.1002/cam4.1544

**Published:** 2018-05-15

**Authors:** Mei‐Xia Wang, Jun‐Ting Ren, Lu‐Ying Tang, Ze‐Fang Ren

**Affiliations:** ^1^ The School of Public Health Sun Yat‐sen University Guangzhou China; ^2^ Mailman School of Public Health Columbia University New York NY USA; ^3^ The Third Affiliated Hospital Sun Yat‐sen University Guangzhou China

**Keywords:** age, breast cancer, mediation, molecular features, prognosis

## Abstract

Young and elderly breast cancer patients are more likely to have a poorer outcome than middle‐aged patients. The intrinsic molecular features for this disparity are unclear. We obtained data from the Cancer Genome Atlas (TCGA) on May 15, 2017 to test the potential mediation effects of the molecular features on the association between age and prognosis with a four‐step approach. The relative contributions of the molecular features (PAM50 subtype, risk stratification, DNAm age, and mutations in *TP53*,*PIK3CA*,*MLL3*,*CDH1*,*GATA3,* and *MAP3K1*) to age disparities in survival were estimated by Cox proportional hazard models with or without the features. Young patients were significantly more likely to have basal‐like subtype, GATA3 mutations, and younger DNA methylation (DNAm) age than middle‐aged patients (*P *<* *.05). Both the young and elderly patients had a significantly increased risk of breast cancer recurrence after adjusted by race, tumor size, and node status (Hazard ratio [HR] (95% confidence interval [CI]): 2.81 [1.44, 5.45], 2.37 [1.45, 3.89], respectively). This increased risk was weakened in the young patients after further adjustments in the molecular features, particularly basal‐like subtype, *GATA3* mutations, and DNAm age (HR [95%CI]: 1.87 [0.81, 4.32]), resulting in 33.5% decreased risk of recurrence. Meanwhile, the adjustments of the molecular features did not alter the recurrence risk for the elderly patients. Compared with middle‐aged patients of breast cancer, poorer prognosis of elderly patients may be caused by aging, while poorer prognosis of young patients was probably mediated through intrinsic characteristics, such as basal‐like subtype, *GATA3* mutations, and DNAm age of the cancerous tissues.

## 1 INTRODUCTION

Breast cancer is known as the most prevalent cancer type and one of the leading causes of cancer death among females worldwide.^1^ The incidence has been increasing worldwide and may continue to rise in the future.^2^ The overall survival time has been prolonged because of the advances in the pharmacological and surgical therapy, but there is still space to improve the prognosis.^3^, ^4^ Plenty of studies have explored the prognostic factors of breast cancer,^4^ and one of the intriguing findings is that the younger and older patients had a poorer outcome than the middle‐aged patients.^5^

Aging may to some extent explain the reasons why the prognosis was worse for older patients but not for younger patients.^6^, ^7^ It was reported that this age disparity in breast cancer survival can be explained by pathologic factors such as hormone receptor status or treatment.^8^, ^9^, ^10^, ^11^ However, the disparity remains even under control of the clinicopathologic features, treatments, or comorbid conditions.^12^, ^13^, ^14^ Therefore, the reasons for the survival disparity of breast cancer need exploration, particularly for the young patients. Clarifying this issue would help provide opportunities for novel molecular‐targeted therapies and improve the prognosis.

We noticed that a series of studies have found certain intrinsic molecular feature changes which were related to age at diagnosis of invasive breast cancer.^15^ For example, *GATA3* mutations in breast tumors occurred more frequently in young patients^16^; significant upregulation of miRNA‐148b was shown in young breast cancer patients^17^; molecular subtype that was determined by gene expression profiling presented age‐associated patterns, in which young patients were more likely to have basal‐like subtype^15^; age‐related DNA methylations were observed in normal breast tissue as well as invasive breast tumors.^18^, ^19^ Furthermore, some of these molecular features have been found to be associated with breast cancer survival.^20^, ^21^, ^22^ Therefore, these molecular features may have effects on the associations between age and breast cancer prognosis.

In this study, we investigated the impacts of the molecular features in young vs elderly breast cancer patients, such as gene and miRNA expression profiles, somatic mutations, and DNA methylation profiling, on survival disparities by age at diagnosis through the breast cancer clinical and molecular data from the Cancer Genome Atlas (TCGA).

## 2 METHODS

### 2.1 Patients

We applied the R/Bioconductor TCGAbiolinks package^23^ (http://bioconductor.org/packages/TCGAbiolinks/) to download all available breast cancer data from Genomic Data Commons (GDC) data portal (https://portal.gdc.cancer.gov/) on May 15, 2017. Meanwhile, molecular data including gene expression, somatic mutations, miRNA expression, and DNA methylation profiling were obtained. Clinical data included survival information, age at diagnosis, race, tumor size, lymph node status, histological type, clinical stage, and statuses of estrogen receptor (ER), progesterone receptor (PR), and human epidermal growth factor receptor 2 (HER2). These hormone receptors were identified by immunohistochemistry. For HER2 status classification, if immunohistochemistry result was equivocal, fluorescent in situ hybridization was used. The endpoint for this study was recurrence‐free survival (RFS), which was defined as the time from diagnosis to the date of recurrence or last follow‐up. Patients with stage IV were excluded because the tumor relapse could not be assessed adequately. A total of 1097 breast carcinoma patients were obtained from TCGA dataset and 41 of them, who were male or diagnosed with stage IV, were excluded. Finally, 1056 eligible breast cancer patients were included in the analysis; 880 patients were successfully followed up; 86 patients experienced breast cancer relapse during the follow‐up period.

### 2.2 Molecular variables

Gene and miRNA expressions were assessed with RNA sequencing data. Breast cancer PAM50 molecular subtype was identified using the genefu R/Bioconductor package. Risk score for prognosis was calculated based on 7 miRNAs and 30 mRNA genes.^24^ Patients were stratified into low‐ or high‐risk groups by median cut 0.033. A total of 729 patients were assessed for the risk stratification. For PAM50 subtypes, 1054 patients were typed by gene expression analysis.

The tumor somatic mutations examined by whole‐exome sequencing from 986 patients were analyzed. Only genes with potential driver mutations in more than 5% of breast cancer patients were included.^25^ Finally, 6 mutated genes were identified, including *TP53*,* PIK3CA*,* MLL3*,* CDH1*,* GATA3*, and *MAP3K1*.

DNA methylation profiling on Illumina 450K and 27K platforms was downloaded. We applied Horvath’ method to predict tumor DNA methylation (DNAm) age,^19^ which is currently the most robust predictor of chronological age.^26^ Briefly, 353 dinucleotide markers were identified as epigenetic clock CpGs from 21,369 CpG probes on the Illumina 27K and 450K platforms with a penalized regression model. Based on the 353 CpGs, DNAm age was estimated as follows.

$$\text{DNAmAge}\;=\;\text{inverse}.F(b_{0}+b_{1}{\mathrm{CpG}}_{1}+\ldots +b_{353}{\mathrm{CpG}}_{353})$$

The mathematical details and software tutorials for DNAm age calculation can be found in the additional files of Horvath.^19^ An online age calculator (https://dnamage.genetics.ucla.edu) is available, by which the DNAm age for the breast cancerous tissues were obtained. Age acceleration (AgeAccel) was calculated by DNA methylation age minus chronological age and analyzed as a binary variable by the cutoff zero.

### 2.3 Statistical analysis

**Figure 1:**
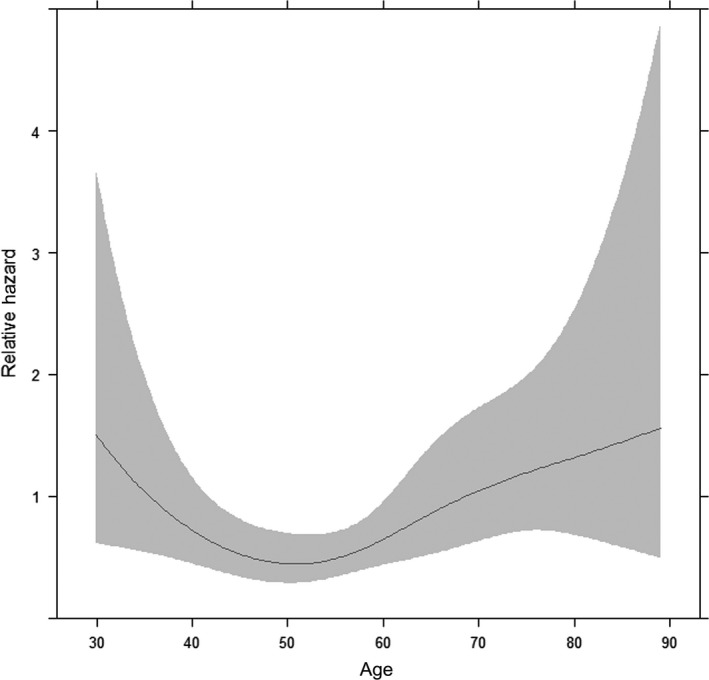
Association between age and relapse‐free survival age was modeled as a continuous variable and fitted in a Cox proportional hazard model using cubic restricted splines with knots at the 5th, 35th, 65th, and 95th percentiles of age, hazard ratio adjusted for race, ER, pathologic stage, HER2; Gray represents the 95 percent confidence interval

According to the result of the association between age and breast cancer recurrence by cubic restricted splines (as shown in Figure 1), under 40 years old was regarded as “young patients”; 60 years and older were defined as “elderly patients”; others were classified as “middle‐aged patients.” Clinicopathological and molecular characteristics of the patients were compared by age group using Kruskal‐Wallis rank test for continuous variables and Pearson’s Chi‐squared test for categorical variables. Fisher’s Exact test was done when the Chi‐squared test was not suitable due to the small size. The associations between age group and molecular characteristics were assessed with multivariate logistic regression models adjusted for race, tumor size, node status, ER status, and HER2 status. All variables were categorical except for DNAm age which was continuous (per 10 years).

The Kaplan‐Meier curve and the log‐rank test were used for the comparison of recurrence‐free survival by age group. Only the patients with full tumor recurrence information were analyzed. To explore the age nonlinear effect on RFS of breast cancer, the age was modeled as a continuous variable and fitted in a Cox proportional hazard model using cubic restricted splines with knots at the 5th, 35th, 65th, and 95th percentiles of age. The relative contribution of each covariable to age disparities in survival was estimated by Cox proportional hazard models with or without the variable of interest. The covariables in baseline model contained age group, race, tumor size, and node status. The influence of molecular covariable on age survival disparities was tested stepwise by adding PAM50 subtype, risk stratification, DNAm age, and 6 gene mutations to the baseline model for adjustment. Hazard ratio (HR) and 95% confidence interval (95%CI) were estimated. The contribution of the covariables was assessed by the equation of (HR_−_ − HR_+_)/HR_0_ *100, in which HR_0_ is the HR from the baseline model, HR_‐_ is the HR from the model without the covariable of interest, and HR_+_ is the HR from the model with the covariable of interest.^27^ Concordance index (c‐index) was applied to evaluate model discrimination. A multinomial propensity score weighting analysis for 3 age groups of patients using R/twang (version 1.5) package (https://CRAN.R-project.org/package=twang) was performed to probed the age‐related difference in breast cancer recurrence, in which race, hormone status, tumor size, node status, basal‐like subtype, DNAm age, 6 gene somatic mutations, and risk score stratification were balanced. *P *<* *.05 was considered to be statistically significant. Statistical analyses were performed using the R‐3.3.3 software.

## 3 RESULTS

### 3.1 Clinicopathological characteristics and the association with age at diagnosis

The mean age of the included 1056 female breast cancer patients was 58.4 years old. Less than 20% were African Americans. Most of the patients were infiltrating ductal carcinoma (71.3%), clinical stage II (58.6%), and ≤2 cm of tumor size (84.3%). More patients were ER or PR positive and HER2 negative. The distributions of these clinicopathological characteristics by age group were shown in Table 1. Young patients were more likely to have infiltrating ductal carcinoma, and be node positive, ER negative, and PR negative. There was a trend of higher pathologic stage, larger tumor size, and more black people in young patients, but no statistical differences were found.

**Table 1:** Clinicopathological characteristics of patients according to age at diagnosis (No. [%])

Characteristics	Total	Age (y)			
		~39	40‐59	60~	*P* value
Race					
African American	173 (18.5)	18 (24.7)	84 (18.3)	71 (16.4)	**.225**
White or others	763 (81.5)	55 (75.3)	375 (81.7)	362 (83.6)	
Histological type					
Infiltrating lobular carcinoma	201 (19.0)	0 (0.0)	84 (17.3)	117 (23.7)	**<.001**
Infiltrating ductal carcinoma	752 (71.3)	68 (90.7)	358 (73.5)	326 (66.2)	
Other	102 (9.7)	7 (9.3)	45 (9.2)	50 (10.1)	
Clinical stage					
Stage I	185 (17.5)	12 (16.0)	73 (14.99)	100 (20.2)	.135
Stage II	619 (58.6)	40 (53.3)	300 (61.60)	279 (56.5)	
Stage III	252 (23.9)	23 (30.7)	114 (23.41)	115 (23.3)	
Tumor size (cm)					
≤2	890 (84.3)	59 (78.7)	420 (86.24)	411 (83.2)	.164
>2	166 (15.7)	16 (21.3)	67 (13.76)	83 (16.8)	
Lymph node status					
Negative	518 (49.2)	27 (36.0)	217 (44.65)	274 (55.7)	**<.001**
Positive	535 (50.8)	48 (64.0)	269 (55.35)	218 (44.3)	
ER					
Negative	235 (23.3)	20 (28.2)	130 (27.90)	85 (18.0)	**<.001**
Positive	775 (76.7)	51 (71.8)	336 (72.10)	388 (82.0)	
PR					
Negative	336 (33.3)	26 (36.6)	172 (36.99)	138 (29.2)	**.033**
Positive	673 (66.7)	45 (63.4)	293 (63.01)	335 (70.8)	
HER2					
Negative	745 (78.1)	49 (76.6)	351 (79.4)	345 (77.0)	.656
Positive/equivocal	209 (21.9)	15 (23.4)	91 (20.6)	103 (23.0)	

ER, estrogen receptor; Her2, human epidermal growth factor receptor 2; PR, progesterone receptor. Bold values mean *P* < 0.05.

### 3.2 Associations between molecular characteristics and the age at diagnosis

Table 2 presents the associations of age at diagnosis with the molecular characteristics of breast cancer. PAM50 subtype, DNAm age, and gene mutations showed age‐related patterns. Older patients had less PAM50 basal‐like subtype and the positive rates of this subtype were 25.3%, 22.6%, and 13.4% among the young patients, middle‐aged patients, and elderly patients, respectively (*P* < .001). In the multivariable model, the elderly patients had a lower adjusted OR for PAM50 basal‐like subtype by 0.51 (95% CI, 0.35 to 0.73) compared with the middle‐aged group. For DNAm age, compared with middle‐aged patients, young patients had a lower adjusted OR of DNAm age (per 10 years) (OR [95% CI], 0.78 [0.66, 0.93]), and the elderly patients had a higher adjusted OR of DNAm age (per 10 years) (OR [95% CI], 1.34 [1.22, 1.48]). A significant association between age at diagnosis and age acceleration in breast cancer was observed, where the elderly patients had a lower age acceleration with an adjusted OR of 0.49 (95% CI, 0.35 to 0.67). As for the gene mutations, the young patients had a higher adjusted OR of *GATA3* mutations (OR [95% CI], 2.19 [1.07, 4.37]) compared with the middle‐aged group; higher OR was found in elderly patients with *MLL3* mutations by an adjusted OR of 1.90 (95% CI, 1.13 to 3.25); *CDH1* mutations showed the similar age‐related pattern to *MLL3* mutations. There was no significant difference by age group in RNA risk stratification.

**Table 2:** Associations of age at diagnosis with the molecular characteristics of breast cancer (No. [%])

Characteristics	40‐59	~39		60~	
	No. (%)	No. (%)	OR (95% CI)^a^	No. (%)	OR (95% CI)^a^
DNAm age					
DNAmAge (per 10 y)	486 (100)	75 (100)	**0.78 (0.66, 0.93)**	492 (100)	**1.34 (1.22, 1.48)**
AgeAccel					
≤0	295 (60.7)	34 (45.3)	1.00 (reference)	348 (70.7)	1.00 (reference)
>0	191 (39.3)	41 (54.6)	**2.21 (1.12, 4.12)**	144 (29.3)	**0.49 (0.35, 0.67)**
Basal‐like subtype					
No	376 (77.4)	56 (77.7)	1.00 (reference)	427 (86.6)	1.00 (reference)
Yes	110 (22.6)	19 (25.3)	1.35 (0.48, 3.78)	66 (13.4)	**0.51 (0.35, 0.73)**
Risk stratification					
Low risk	122 (35.4)	26 (42.6)	1.00 (reference)	136 (42.1)	1.00 (reference)
High risk	223 (64.6)	35 (57.4)	0.61 (0.33, 1.12)	187 (57.9)	0.72 (0.50, 1.03)
*TP53* mutation					
No	259 (58.5)	47 (68.1)	1.00 (reference)	309 (70.9)	1.00 (reference)
Yes	184 (41.5)	22 (31.9)	**0.48 (0.23, 0.99)**	127 (29.1)	**0.63 (0.44, 0.92)**
*PIK3CA* mutation					
No	306 (69.1)	51 (73.9)	1.00 (reference)	275 (63.1)	1.00 (reference)
Yes	137 (30.9)	18 (26.1)	0.69 (0.33, 1.33)	161 (36.9)	1.24 (0.90, 1.73)
*GATA3* mutation					
No	375 (84.7)	52 (75.4)	1.00 (reference)	395 (90.6)	1.00 (reference)
Yes	68 (15.3)	17 (24.6)	**2.19 (1.07, 4.37)**	41 (9.4)	**0.39 (0.24, 0.64)**
*MLL3* mutation					
No	412 (93.0)	66 (95.7)	1.00 (reference)	380 (87.2)	1.00 (reference)
Yes	31 (7.0)	3 (4.3)	**0.09 (0.01, 0.45)**	56 (12.8)	1.45 (0.94, 2.23)
*CDH1* mutation					
No	389 (87.8)	68 (98.5)	1.00 (reference)	352 (80.7)	1.00 (reference)
Yes	54 (12.2)	1 (1.5)	**0.26 (0.02, 1.28)**	84 (19.3)	**1.90 (1.13, 3.25)**
*MAP3K1* mutation					
No	414 (93.5)	67 (97.1)	1.00 (reference)	386 (88.5)	1.00 (reference)
Yes	29 (6.5)	2 (2.9)	0.41 (0.06, 1.45)	50 (11.5)	1.61 (0.96, 2.73)

AgeAccel, Age Acceleration; DNAm Age, DNA methylation age, DNAmAge minus Chronological Age. Risk stratification was grouped by median value of risk score, patients were predicted as high risk group if risk score larger than 0.033, others were predicted as low risk group.

Odds ratio adjusted for race, tumor size, node status, ER status, HER2 status. Bold values mean *P* < 0.05.

### 3.3 Associations of molecular characteristics with breast cancer recurrence

The associations between the molecular characteristics and recurrence‐free survival for breast cancer were shown in Table 3. Younger DNAm age or low DNAm age acceleration, PAM50 basal‐like subtype, and high RNA risk were significantly associated with an increased risk of tumor recurrence. No significant association between gene mutations and breast cancer relapse was found.

**Table 3:** Associations of molecular characteristics with breast cancer recurrence

Characteristics	n/event	HR^a^ (95% CI)
DNAmAge (per 10 y)	819/81	**0.88 (0.76, 0.96)**
Age Acceleration (per 10 y)	819/81	**0.87 (0.75, 0.95)**
PAM50 basal‐like subtype		
No	714/64	1.00 (reference)
Yes	164/22	**1.70 (1.03, 2.82)**
Risk stratification		
Low risk	222/21	1.00 (reference)
High risk	352/39	**1.83 (1.03, 3.22)**
*TP53* mutation		
No	511/45	1.00 (reference)
Yes	270/26	1.18 (0.71, 1.98)
*PIK3CA* mutation		
No	513/49	1.00 (reference)
Yes	268/22	0.96 (0.56, 1.62)
*GATA3* mutation		
No	678/62	1.00 (reference)
Yes	103/9	0.82 (0.37, 1.81)
*MLL3* mutation		
No	706/65	1.00 (reference)
Yes	75/6	1.08 (0.46, 2.54)
*CDH1* mutation		
No	655/58	1.00 (reference)
Yes	126/13	0.81 (0.40, 1.64)
*MAP3K1* mutation		
No	719/49	1.00 (reference)
Yes	62/2	0.46 (0.11, 1.84)

AgeAccel, Age Acceleration; DNAm Age, DNA methylation age, DNAmAge minus Chronological Age. Risk stratification was grouped by median value of risk score, patients were predicted as high risk group if risk score larger than 0.033, others were predicted as low risk group.

Hazard ratio adjusted for race, tumor size, node status, age groups. Bold values mean *P* < 0.05.

### 3.4 Age effect on breast cancer recurrence and the impact of molecular features

**Figure 2:**
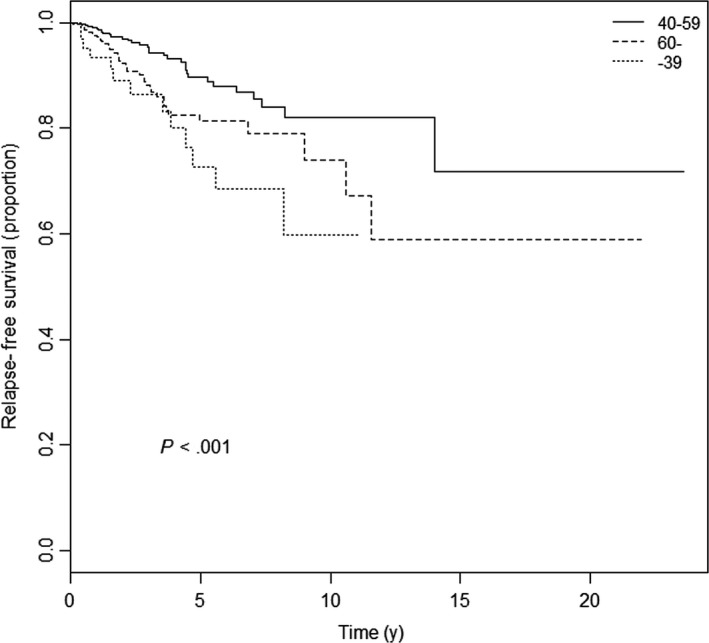
Kaplan‐Meier plot for relapse‐free survival according to age at diagnosis of breast cancer

When age was modeled as a continuous variable and fitted in the multivariable Cox proportional hazard model using cubic restricted splines to estimate age nonlinear effect, an obvious nonlinear association between age and RFS was shown (*P *=* *.012, Figure 1). The ages of 40 and 60 years were likely to be reasonable cutoff values according to the association between age and survival prognosis. Based on these age cutoff values, Kaplan‐Meier analysis showed that the young and elderly breast cancer patients had a shorter time to relapse than the middle‐aged patients (Figure 2, *P* < .001).

We then explored the contribution of molecular features to age‐related disparities in breast cancer recurrence (Table 4). In the univariate model, the young patients had a 2.96‐fold increase in the risk of breast cancer recurrence compared with the middle‐aged patients (HR [95% CI]: 2.96 [1.54, 5.67]). After adjusted by race, tumor size, and node status, the HR was slightly decreased (Model 2 in Table 4). We further adjusted stepwise, the PAM50 subtype (Model 3), risk stratification (Model 4), DNAm age (Model 5), and gene mutations (*GATA3*,* PIK3CA*,* MLL3*,* CDH1*,* TP53*, and *MAP3K1*) (Model 6‐11), it turned out that the strength of the association between breast cancer recurrence and young patients over middle‐aged patients was gradually weakened. In Model 5 to model 11, the association was not statistically significant and all the molecular features overall decreased 33.5% ([2.81‐1.87]/2.81) of the recurrence risk among the young patients compared with the middle‐aged patients. For the elderly patients, however, the poor prognosis persisted after the adjustment of these molecular characteristics.

**Table 4:** Associations between age at diagnosis and breast cancer recurrence

Models	n/event	Hazards ratio (95% CI)			c‐index
		40‐59	~39	60~	
Model 1	880/86	1.00 (reference)	2.96 (1.54, 5.67)	1.93 (1.21, 3.08)	0.61
Model 2	822/81	1.00 (reference)	2.81 (1.44, 5.45)	2.37 (1.45, 3.89)	0.68
Model 3	537/57	1.00 (reference)	2.51 (1.28, 4.89)	2.49 (1.51, 4.10)	0.71
Model 4	537/57	1.00 (reference)	2.22 (1.06, 4.65)	1.84 (1.03, 3.37)	0.69
Model 5	535/57	1.00 (reference)	1.97 (0.94, 4.17)	2.03 (1.11, 3.74)	0.71
Model 6	503/52	1.00 (reference)	1.93 (0.85, 4.39)	2.11 (1.13, 3.40)	0.70
Model 7	503/52	1.00 (reference)	1.89 (0.84, 4.29)	2.11 (1.13, 3.96)	0.70
Model 8	503/52	1.00 (reference)	1.84 (0.81, 4.20)	2.15 (1.15, 4.04)	0.70
Model 9	503/52	1.00 (reference)	1.93 (0.85, 4.34)	2.12 (1.13, 3.97)	0.70
Model 10	503/52	1.00 (reference)	1.85 (0.82, 4.23)	2.12 (1.13, 3.98)	0.70
Model 11	503/52	1.00 (reference)	1.89 (0.83, 4.28)	2.17 (1.16, 4.07)	0.70
Model 12	503/52	1.00 (reference)	1.87 (0.81, 4.32)	2.13 (1.13, 4.03)	0.70

Model 1, unadjusted; Model 2, adjusted by race, tumor size, node status; Model 3, further adjusted by PAM50 subtype; Model 4, further adjusted by risk stratification; Model 5, further adjusted by DNAm age; Model 6‐11, further separately adjusted by GATA 3, PIK3CA, MLL3, CDH1, TP53, MAP3K1; Model 12 adjusted all the variables.

**Figure 3:**
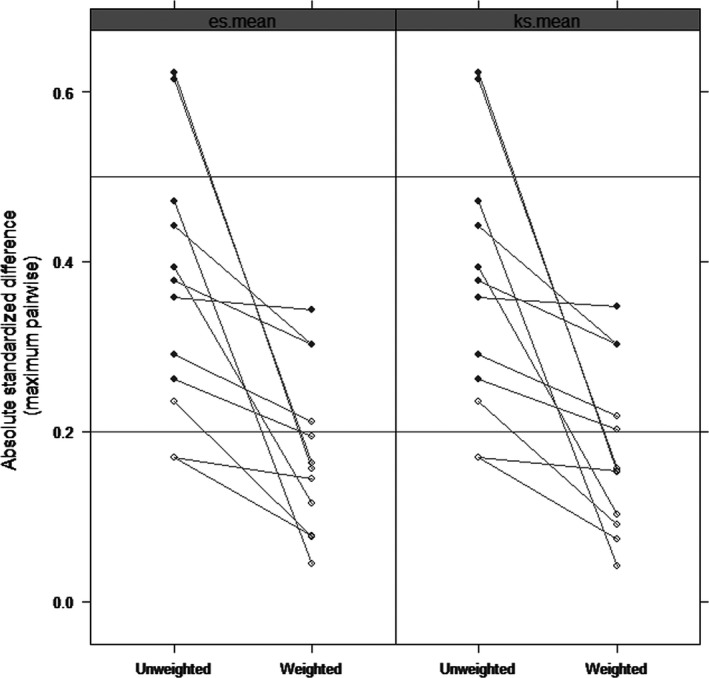
Comparisons of the absolute standardized mean differences (ASMD) by es.mean and ks.mean methods between the age group on the covariates before and after propensity score weighting. The covariates included race, tumor size, node status, PAM50 molecular subtype, risk stratification, DNAm age, and somatic mutations. The statistically significant difference is indicated by the solid circle. The decreases of ASMD after weighting indicates good covariate balance

Finally, we performed a propensity score analysis, balancing race, tumor size, node status, PAM50 molecular subtype, risk stratification, DNAm age, and gene mutations among the 3 age groups of patients (Figure 3). The propensity score‐adjusted Cox regression showed the same results that the young patients had no significant difference in tumor relapse when comparing the middle‐aged patients (HR [95% CI], 1.71[0.59, 4.37]), while the elderly patients remain a high hazard ratio for RFS by 2.04 (95% CI, 1.07 to 3.88).

## 4 DISCUSSION

In this study, we confirmed that young and old age at diagnosis were associated with an unfavorable clinical outcome in breast cancer compared with the middle‐aged patients, which was in line with most of the previous studies.^5^, ^11^, ^28^ However, a few of previous studies did not find the nonlinear association between age and survival prognosis.^29^, ^30^ Meanwhile, several studies proposed that age was not an independent prognostic factor for breast cancer.^31^, ^32^ We noticed that these previous studies applied various cutoff values of age, such as that young age was defined as under the ages of 30, 35, 40, 45, or 50,^33^ which might contribute to the inconsistent results. We firstly applied cubic restricted splines to accordingly define “young patients,” “middle‐aged patients,” and “elderly patients,” which was consistent with Jianfei’s definition determined by X‐tile program.^33^

It may be understandable and reasonable that elderly breast cancer patients had a worse prognosis than middle‐aged patients due to the impaired capacity with aging or undertreatment.^7^, ^10^, ^34^, ^35^ As for the worse prognosis of young patients, previous studies have attributed it to tumor invasiveness, hormone status, tumor subtype, and treatment.^8^, ^36^, ^37^, ^38^, ^39^ We also similarly observed that these tumor characteristics contributed to the poor prognosis for young patients to some extent. Moreover, this study showed that the adjustments of the molecular features substantially decreased the strength of the association between young age and survival prognosis (33.5%), suggesting that the molecular characteristics also likely played roles for the poor prognosis of young patients.

The molecular characteristics with significant impacts found in this study included PAM50 subtype, DNAm age, and *GATA3* mutations. It was shown that PAM50 subtype decreased the strength of the association between age and prognosis of breast cancer, which was in line with the results from previous studies using the molecular subtype determined by immunohistochemistry routinely applied in clinical practice.^40^, ^41^ However, there were also negative results that the immunohistochemical subtype did not influence the association of age with the prognosis^42^, ^43^ One of the reasons for this inconsistency was that the immunohistochemical subtype only roughly resembles the intrinsic properties.^44^, ^45^ PAM50 molecular subtype determined by 50‐gene expression was able to more accurately reflect the distinctive expression pattern of breast cancer than that with the routine clinical method,^44^, ^45^ particularly for low ER staining.^46^

We found that younger DNAm age decreased the strength of the association between chronological age and prognosis of breast cancer. It can be explained partly by the association that younger tumor DNAm age had a poor prognosis, which has also been reported in several other cancers.^26^ Younger DNAm age was associated with the potential to promote malignant transformation and propagation^19^ resulting in the increase in breast cancer relapse; it was also related to higher frequencies of genetic mutations which increased the invasiveness for young breast cancer patients.^19^, ^26^ We did not find the impact of DNAm age in the elderly, which may be explained to some extent by that the association between DNAm age and chronological age dramatically declined with increased age.^47^

We further found that *GATA3* mutations might play a role in the poor prognosis for young patients. A total of 140 somatic mutations in *GATA3* were detected in 13.7% of 986 patients in the TCGA database (updated May 15, 2017). Among them, more than two‐thirds (67.8%) were frame shift mutations which resulted in proteins with extended C‐terminus and induced peptidyl‐tyrosine modification and cancer progression.^48^ It was also reported that mutations of *GATA3* in breast cancer cells were related to reduced DNA binding ability and increased cell proliferation, resulting in endocrine resistance.^49^, ^50^ We found that the old patients had much less frame shift mutations in *GATA3* than young patients, which may be the reason that *GATA3* mutations had no effects on the associations between age and prognosis of breast cancer.

We conducted a four‐step approach proposed by Baron and Kenny^51^ to test the potential mediation effects of the molecular features on the association between age and prognosis. First, age at diagnosis affected molecular features in logistic regression; second, the molecular features were associated with survival prognosis in Cox’s regression model; third, associations between age and the prognosis were examined in Cox’s regression model with and without adjustment of the molecular features (potential intermediate variables); finally, the significances and changes of hazard ratios derived from the models with or without the adjustments were taken as the evidence of mediation. It should be noted that this method still only statistically proved the mediation effects and the causal interpretation remains to be explored, particularly by biological experiments.

There were also some other potential limitations in this study. First, breast cancer‐specific fatality was not included as the outcome due to the lack of death causes in TCGA database. However, recurrence‐free survival may more accurately reflect breast cancer‐specific survival. Second, we did not have the information about potential confounders such as sociodemographic and therapy, but this missing information may not change the mediating effects of the molecular features on age‐related prognosis because it was reported that age was an independent prognostic factor regardless of sociodemographic and therapy.^5^, ^12^ Third, the small sample size may reduce the statistical power, which was probably the reason for the results that there were nonsignificant effects of RNA risk score and the mutations of other genes (*TP53*,* PIK3CA*,* MLL3*,* CDH1*,* MAP3K1*) on the association between age and the prognosis of breast cancer.

In conclusion, we demonstrated that tumor molecular features could contribute to the known poor prognosis for young breast cancer patients (accounted for 33.5% of disparities in poor prognosis) but not for the elderly. These results suggested that the intrinsic molecular features likely played a fundamental role in the poor prognosis for young patients. The molecular‐targeted therapies among young patients can promisingly improve survival prognosis.

## CONFLICT OF INTEREST

The authors declare that they have no conflicts of interest.
